# Microbial Barrier Properties of Cotton Fabric—Influence of Weave Architecture

**DOI:** 10.3390/polym12071570

**Published:** 2020-07-15

**Authors:** Beti Rogina-Car, Stana Kovačević, Ivana Schwarz, Krste Dimitrovski

**Affiliations:** 1 Department of Clothing Technology, Faculty of Textile Technology, University of Zagreb, Prilaz baruna Filipovića 28a, 10000 Zagreb, Croatia; 2 Department of Textile Design and Management, Faculty of Textile Technology, University of Zagreb, Prilaz baruna Filipovića 28a, 10000 Zagreb, Croatia; stana.kovacevic@ttf.unizg.hr (S.K.); ivana.schwarz@ttf.unizg.hr (I.S.); 3 Faculty of Natural Sciences and Engineering, University of Ljubljana, Aškerčeva 12, 1000 Ljubljana, Slovenia; krste.dimitrovski@gmail.com

**Keywords:** microbial barrier, weave architecture, pores number, pores size, yarns floating

## Abstract

The subject of the paper focuses on the effect of weave architecture on microbial barrier properties of woven fabrics or more precisely on identifying crucial elements of weave architecture that dominantly influence bacteria penetration in dry condition. For that purpose, 12 samples of cotton fabrics were woven and examined. In their structure, all samples had the same yarns (36 tex) in warp and weft, same densities of warp (24 yarns/cm), two weft densities (24 and 20 yarns/cm) and six different basic weave structures. Microbial barrier permeability was determined according to a previously developed test method in cooperation with University Hospital Center Zagreb. Bacterial endospores of apathogenic species of the genus Bacillus: *Geobacillus* *stearothermophilus* and *Bacillus atrophaeus* were used. The effect of weave pattern on microbial barrier properties was significant. Weave patterns, decisively determined the number of influencing pores and its sizes in woven fabrics, as well as the yarn floating which jointly almost perfectly correlated with bacteria penetration through the woven fabric. Multiple linear regression of pore numbers and floating threads produced equations which correspond in 99% to the measuring results for densities 24/24 and 24/20, and more than 98% considering both densities of the set. Among compared weave patterns, satin weave had significantly lower permeability of microorganisms (six–seven times) than basket weave (the highest), for both densities.

## 1. Introduction

Requirements of the microbial barrier are important in the application of textile materials for protective clothing, which is primarily used in medicine in operating rooms (surgical gowns, caps, masks, surgical drapes, bedding and covers for medical tools as well as other items in operating rooms), in intensive care units, especially in sterilization for packaging medical supplies, in departments (protective clothing for medical staff), etc. Textile materials with microbial barrier properties are also used in other sterile areas such as production, preparation, packaging of groceries and medications, etc. [1]. Due to better, more efficient and economical protection, the woven fabric has an advantage over other textile materials. Fabric structure ensures that even after multiple use and washing, its properties remain satisfactory, such as dimensional stability, strength, durability, abrasion resistance, breathability, etc., while other materials do not achieve and retain all these properties at the same time. Fabric structural parameters such as weave, warp and weft density, linear density, yarn density and yarn twist determine permeability and possible infiltration. However, attempts to correlate these parameters with the permeability of woven fabrics are rare. The anisotropic flow behavior of isotropically formed textiles is explained by using the crimp of warp and weft threads. Textiles with a higher crimp ratio (warp/weft) showed a higher anisotropy in permeability [1,2]. 

### 1.1. Woven Fabrics in Function of Medical Textiles

Textile materials are known to be an excellent substrate for the growth of bacteria and fungi under appropriate moisture and temperature conditions [3,4]. Contaminated fabrics allow pathogens to be transmitted endogenously and exogenously. This increases the possibility of spreading various life-threatening infections among medical staff and patients in medical institutions, consuming contaminated food, etc. Critical zones when using fabrics with microbial barrier properties include areas of direct contact with sources of contamination. Microorganisms can be present in aerosol form, in a liquid, and therefore pass through the fabric to the body. Previous studies on the barrier properties of fabrics are based on liquid permeability and are mainly related to surgical gowns and drapes (e.g., mason jar test and fluid pressure penetration). Liquid penetration tests use water and synthetic or human blood [3,4,5,6]. A sterile barrier can be defined as a material placed between a sterile and contaminated area in order to protect against the penetration of microorganisms through the fabric [7,8,9]. A study by Sattar [10] revealed greater and faster mobility of bacteria in moist conditions than in dry conditions. Bacterial transmission is increased by rubbing into a fabric. A study by Cervantes et al. [11] revealed that the bacterial contamination of professional medical uniforms occurs within hours of wearing a clean uniform. A study by Wierner-Well et al. describes a survey of 135 medical professionals (doctors and nurses). The results revealed that more than 60% of their uniforms were colonized by potentially pathogenic bacteria, including drug-resistant microorganisms [12]. 

Medical textiles play a special role of protection in packaging and sterilization. At the same time, there are almost no methods in literature describing penetration of bacteria through the fabrics in dry conditions. Such a method was developed within cooperation of the Faculty of Textile Technology and the Medical Faculty in Zagreb, Croatia [13]. A detailed description of the method was presented in the Journal Cellulose [9] and results of investigation were presented in several scientific papers [9,14,15,16]. The only standardized method which is used for examining resistance of dry textiles against penetration of microorganisms is according to standard EN ISO 22612 and uses contaminated talc that is put on the material under the pressure and exposed to oscillation for certain time. After that the permeability is determined with counting of colonies on the agar plate [17]. The difference between the mentioned standard and the newly developed method of microbial barrier testing i.e., microorganism’s permeability is in the direct application of dry microorganisms to a dry sterilized textile sample. The bacterial spores of the genus Bacillus: *Geobacillus stearothermophilus* and *Bacillus atrophaeus* are used as the only dry-type microorganisms. Furthermore, with the standardized method, the microorganisms were infiltrated into contaminated talc. With continuous mechanical vibration and pressing with weights, microorganisms pass through the woven fabric forcibly and more easily. In the newly developed method, the microorganisms pass through the fabric without pressure in a time period of 24 hours. That simulates conditions like the actual application in the hospital environment from packing packages in sterilization to healthcare professional uniforms in hospital wards.

The system of microbiological barrier must allow the process of sterilization (permeability of air and sterilization media), prevent penetration of microorganisms, and keep the sterility until the moment of use. Packaging protects sterilized material from contamination during different condition and time of handling, transportation, and storing [18,19]. 

### 1.2. Structural and Permeability Properties of Woven Fabrics 

When producing woven fabrics, three to five parameters must be defined—yarn fineness in warp and weft and yarn densities in warp, weft, and weave. Some parameters are already predetermined, such as type and fineness of yarns of woven fabric. The parameters to be defined are limited to interval of selection of densities of the selected weave. All the other physical (crimp, thickness, physical density, mass of unit area, porosity, etc.), mechanical (breaking strength and elongation, tensity, forces of rupture, resistance to abrasion, etc.) and permeability properties (permeabilities of gasses, liquids, light, sound, energy, water vapor, bacteria, etc.) are affected by previously mentioned selection. 

Although the fineness of yarns and densities in woven fabrics can be expressed and described with a single number, that is not the case with weaves. The characteristics of weaves depend on several important elements such as: weave unit size, square or rectangular shape, number of interlacing points in the unit, length of floating of yarns, number of consecutive yarns that interlace identically, domination of warp or weft, or equal domination of warp and weft yarns on the face and back side of fabric. Everything listed above in conjunction with the differences in selected densities and fineness of yarns, make a large pallet of woven fabrics with different permeability properties. It is also important to acknowledge that certain technological parameters (the number of yarns in reed dent) and after-treatment significantly contribute to the permeability properties. The number of yarns in reed dent, in conjunction with the weave parameters, often group the pores in at least two groups. The treatment of fabric that follows usually diminish existed differences among them. 

Fabric is defined as a porous material made of fibers and air [20]. Fabric porosity is determined by the size, distribution, and shape of pores and it depends on fiber and yarn properties and structural fabric properties, such as weave pattern, density, and thread fineness [21,22,23,24]. The paper by M. Havlová [24] explains that plain-weave fabrics have inter-yarn pores of approximately the same shape. However, pore size varies, which has a significant impact on air permeability. Many researchers investigated it. Some of them are mentioned in this paper [25,26,27,28,29,30,31,32,33]. Fabric permeability is observed at the meso level (order of magnitude 10^−3^–10^−2^ m). Such an observation of the structure involves the analysis of the basic elements of the weave pattern (weave unit, interlacing point) or the interaction of yarns. The impact of fabrics constructional parameters on pore size is shown in Table 1 [34].

Weave pattern is often neglected when choosing fabrics for protective clothing; while more attention is given to mass per unit area, raw material composition, thickness, and color. Fabric weave structure is often viewed as fabric surface appearance and does not correlate with fabric properties. 

The main objective of this work is primarily identifying the main elements of weave architecture that dominantly influence bacteria penetration in dry conditions and thus determine the impact of weave pattern on penetration of bacteria as unexplored area of crucial importance for the protection and preservation of human health in contaminated areas.

## 2. Materials and Methods 

### 2.1. Materials

Fabric samples were woven using the same warp with the same warp thread density (24 ends/cm) and two weft thread densities (24 and 20 picks/cm), and in 6 weave patterns: satin weave (S), twill weave (T), plain weave (P), warp rib weave (R1), weft rib weave (R2), and basket weave (B). The Picanol Omni Plus 800 air jet weaving machine belonging to the Textile Company Čateks d.d. Čakovec, Croatia was used to weave per 100 m of the fabric in each weave pattern.

The warp threads were drawn in the reed as 2 threads per dent and the finesses of the reed was 12 dents/cm. The same yarn was used both for the warp and for the weft: 100% cotton yarn with a count of 36 tex and 505 turns/m, and S twist direction of yarn. After finishing the weaving, the fabrics were washed to remove the size coating from the warp yarns, and then dried in the dryer during which the weft was straightened. These woven fabrics can be used alone or in a composite for technical purposes, clothing, etc. 

### 2.2. Methods

In the course of investigations, the attempts were made to find correlations between physical and structural characteristics of fabrics as a function of weave pattern, such as: mass per unit area, thickness, specific density, porosity and its parameters (number, size in distribution of pores), air permeability and rate of bacteria from one side of the fabric to the other side. A microscopic images of the samples were made for visual assessment of their transparency, number of pores and their size. 

The constructional parameters of fabrics were tested according to the standards: ISO 2060 (yarn count–yarn linear density) and ISO 7211-5 (linear density of yarn removed from fabric) and ISO 1049-2 (fabric density). Physical characteristics were tested according to ISO 3801 (fabric mass) and ISO 5084 (fabric thickness) and the results are shown in Table 2 and Table 3 [34,35,36,37,38]. The physical density (ρ_fabric_) and fabric porosity (P) were calculated based on the measured surface mass and thickness, Equation (1) and Equation (2) [39].

(1) $$\;\rho_{fabric}=\frac{M}{D\cdot 1000}\;\left[g/cm^{3}\right]$$

where: ρ*_fabric_*—physical density of a fabric (g/cm^3^), *M*—mass per square unit (g/m^2^), and *D*—thickness (mm).

(2) $$P=\left(1-\frac{\rho_{fabric}}{\rho_{material}}\right)\;\cdot 100\;\left[\%\right]$$

where: *P*—fabric porosity (%), ρ*_fabric_*—physical density of a fabric (g/cm^3^), and ρ*_material_*—fiber density (g/cm^3^). The value of 1.52 g/cm^3^ was taken for the density of cotton fibers. 

#### 2.2.1. The Yarn Linear Density

The yarn linear density is determined by the skein method according to ISO 2060. The test is carried out on 10 skein, each of 100 m long yarn, which are conditioned and weighed. From the obtained data, the fineness in tex is calculated [34]. The linear density of yarn removed from the fabric is determined according to ISO 7211-5. Ten yarns are removed from the fabric in the warp and weft direction. The length and mass of the yarn are determined. Before determining the length, the yarn is loaded with 0.5 cN / tex to flatten. From the obtained data, the yarn linear density in tex is calculated [36].

#### 2.2.2. Warp and Weft Density (i.e., Number of Threads Per Unit Length)

In accordance with standard EN 1049-2: 2003, the number of warp and weft threads per unit length in fabrics are determined by counting the threads per length of 10 cm in the warp and weft direction. Ten measurements are taken per sample in both directions and the arithmetic mean (in threads/cm) is calculated [37].

#### 2.2.3. Woven Fabric Mass Per Unit Area

In accordance with ISO 3801:1977, the mass per unit area is the mass of the square meter of the woven fabric expressed in grams [g/m^2^]. Samples of dimensions 100 cm^2^ are conditioned and weighed on an analytical balance with a precision of 0.01 g [38].

#### 2.2.4. Woven Fabrics Thickness

The thickness of the woven fabric is measured with a thickness meter. The fabric sample is placed between two rigid tiles of known thickness. Tile thickness is subtracted from the total thickness value. Ten measurements are taken per sample at different locations and the arithmetic mean is calculated, according to ISO 5084: 2003 [40].

#### 2.2.5. Air Permeability

The air permeability of samples was measured in line with the ISO 9237:1995 (E) standard on an air permeability tester Airtronik (Mesdan, Italy). Air permeability was measured on five different places of samples at 100 Pa according to the standard and for the purpose of determining the average equivalent diameter of pores at the pressures of 10, 15, 20, 25, and 30 Pa [41].

The equivalent average pore diameter was calculated using the Hagen–Poiseuille equation (3) under the assumption that the curve air flow—pressure is linear when applying low pressure [42,43,44].

(3) $$Q=\frac{\pi \;\cdot \;d^{4\;}\cdot \;n\;\cdot \;\Delta p\;}{128\;\cdot \;\mu \;\cdot \;l}=A\cdot \;\Delta p\;\left[cm^{3}/s\right]$$

where: *Q*—volume flow rate (cm^3^/s, at surface of 1 cm^2^), *d*—diameter of pores (cm), *n*—number of pores, µ–kinematic coefficient of viscosity (Pas) in our case 1.8369 × 10^−5^, *l*—thickness of fabric/length of pores (cm), and Δ*p*—pressure drop (Pa).

Equivalent average diameter of pores is calculated by Equation (4), where number of pores n is derived from warp and weft density product.

(4) $$\;d_{e}=\sqrt[{4}]{\frac{128\;\cdot \;A\;\cdot \;\mu \;\cdot l}{\pi \;\cdot n}}$$

#### 2.2.6. Microbial Barrier Properties 

**Figure 1:**
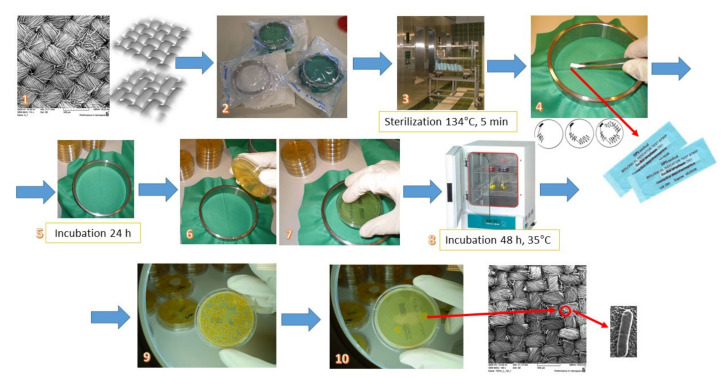
Schematic representation of microbial barrier permeability testing: 1—fabric (sample); 2—samples packaged for sterilization; 3—sterilization; 4—application of microorganisms; 5—incubation; 6 and 7—taking prints; 8—incubation; 9 and 10—enumeration of microorganisms (colony forming units (CFUs)) on the face and back side of the fabric.

Prior to the testing, the textile samples were fixed on an O-ring device and then placed in transparent packaging. The packaged samples were subsequently exposed to sterilization at 134 °C for 5 min, after which the packaging was opened in a sterile environment to prevent contamination. In aseptic conditions, the spores were rubbed in equal motions on the front side of the tested samples (standard conditions at a temperature of 20 ± 2 °C and a relative humidity of 65% ± 5%). The procedure was then repeated in the same order with the biological indicator stick reversed. A print was taken, after 24h using a CT3P agar print plate (bioMe´rieux SA, Marcy I’Etoile, France), first from the back side, and then the front side, with a new plate. Agar plates were incubated for 72 h at 35 °C, after which colony forming units (CFUs) were counted, Figure 1 [9,13].

**Figure 2:**
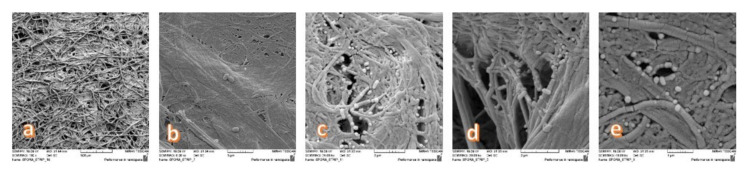
Bacterial endospore *Geobacillus Stearothermophilus* and *Bacillus Atrophaeus* (MesaLabs, Inc. USA) images were taken with a scanning electron microscope (SEM). Magnification: (**a**) 100×; (**b**) 6000×; (**c**) 24,000×; (**d**) 30,000×; (**e**) 40,000×.

The most resistant forms of microorganisms of the bacterial endospore of the apathogenic species of the genus *Bacilllus: Geobacillus Stearothermophilus 10^5^ and Bacillus Atrophaeus 10^6^* were used for testing. They were taken with a field emission scanning electron microscope Mira Tescan (FE SEM, Mira II LMU, Tescan, Brno, Czech Republic), Figure 2. The Bacterial spores were coated with a conductive Ag/Pt layer and scanned under the conditions of high voltage (HV 10.00 kV).

The specificity of the work lies in the use of spores that can survive in a dry environment, while suspensions of different types of microorganisms are used in similar assessments of the microbial barrier. The use of suspension moisturizes the fabric and changes permeability. By using these spores, it was possible to keep the fabric dry and to test the properties in conditions similar to the actual use. 

## 3. Results and Discussion

**Figure 3:**
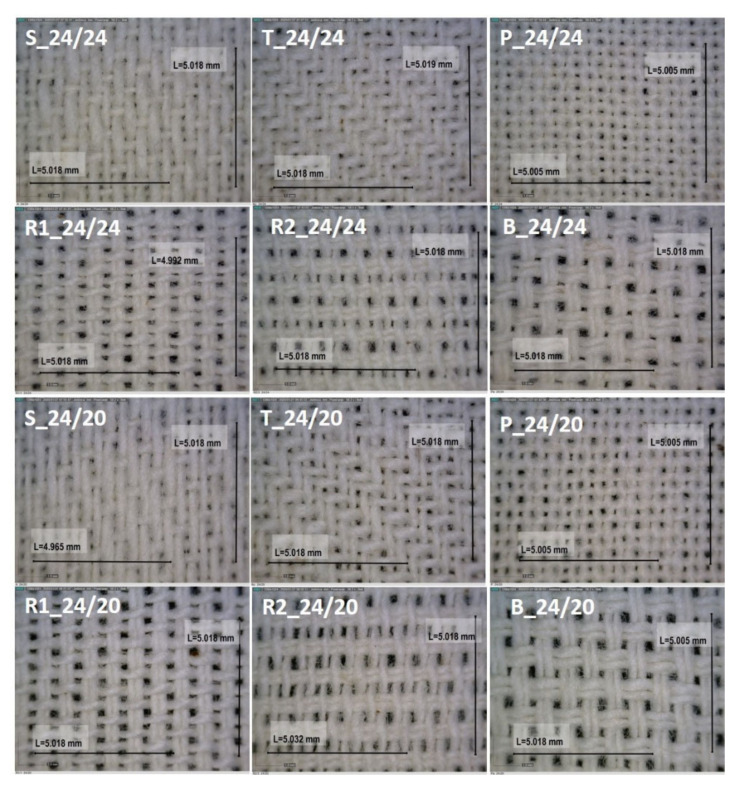
Microscopic image of the surface of fabrics and pores for each weave pattern and both thread densities, Magnification: 60×.

Microscopic images of examined samples are shown in Figure 3. Figure 3 shows that visual transparency of satin and twill weave is smaller compared to the rest of samples. Those samples have longer floating of yarns in their structure.

Figure 3 shows that the pores in the group of 24/24 densities are expectably quadratic and smaller in comparison with the pores of group 24/20 density which are rectangular and bigger. In all of the samples the denting has an important role. The warp yarns in the same dent of reed were closer than those in different dent of reed. This caused the formation of two categories of pores in five samples (except R2)—pores with smaller width between warp yarns in 1 dent and pores with larger width between warp yarns in other, bordering dent. Weave with its particularities significantly contribute to the number, size, and shape of pores. In the basket, every pair of adjacent yarns are equally woven, meaning the pores between them are considerably small compared to the pores that are woven in different way. In warp and weft ribs, which have the same structure but are turned by 90°, this is true only in one structure of yarn (warp or weft).

It was observed that, in sample R2 the insertion to reed causes three categories of pores according to the size and shape. Assuming that the air and bacteria only or mostly pass through pores of larger diameter (smaller pores are disregarded), it was decided to use two numbers of pores: product of actual density of warp and weft and product of warp and weft yarn that are differently woven.

**Figure 4:**
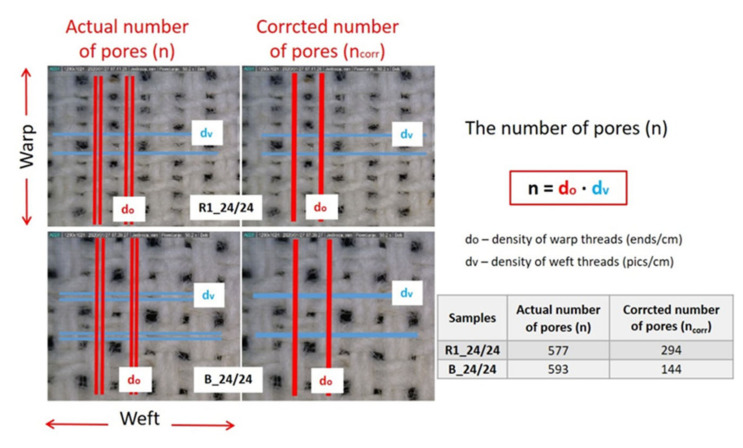
Actual number of pores and corrected number of pores.

This means that in ribs the number of pores will reduce by half, and in basket the number of pores will reduce by one quarter, considering actual number of pores between yarns, Figure 4. 

The constructional and physical parameters of examined fabrics are shown in Table 2 and Table 3. [36,37,38,40].

Calculated values of specific density (ρ*_fabric_*, g/cm^3^) and porosity (P, %), set number of pores (product of set densities), actual number of pores 1 (product of measured warp and weft density) and corrected number of pores 2 (product of yarns that interlace differently), and equivalent average diameter (EAD) of pores 1 (in calculation used actual number of pores) and equivalent average diameter of pores 2 (in calculation used corrected number of pores) are presented in Table 4.

**Figure 5:**
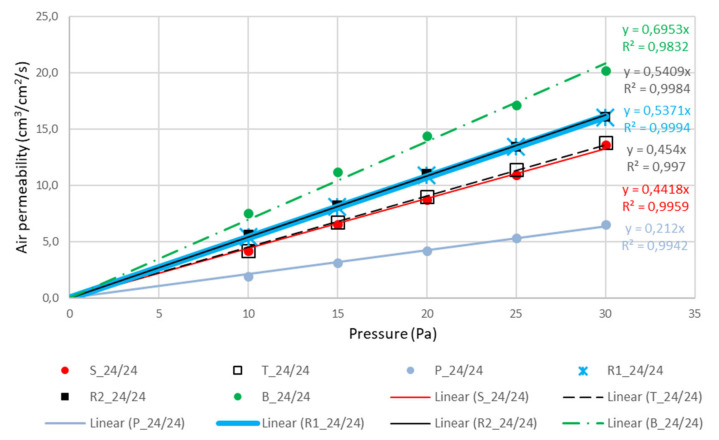
Linear regression of curves air permeability—pressure for the samples in densities 24/24.

**Figure 6:**
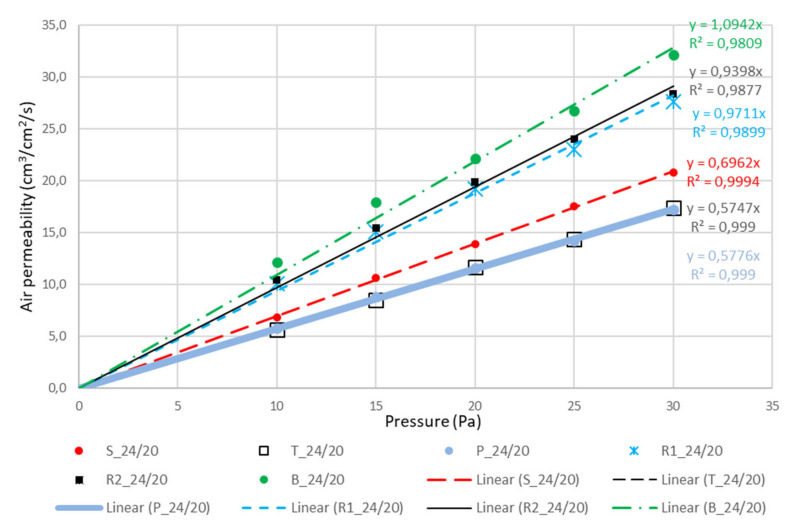
Linear regression of curves air permeability—pressure for the samples in densities 24/20. This time the curves for plain and twill are covering each other.

There is no need for commenting measured constructional, measured physical and calculated results presented in Table 2, Table 3 and Table 4, because all of the presented values are in expected intervals. Only the corrected number of pores dramatically changed/increased the equivalent average diameter of pores for rib weaves for about 25 and for basket 56 micrometers (samples 24/24) and 28–65 micrometers (samples 24/20). The range of equivalent diameter of pores was almost the same in all cases: basket, rib weaves (almost the same), satin, twill, and plain. Because equivalent average diameter is calculated from the measurements of air permeability under low pressure (Equations (1) and (2)), for which the results are presented in Table 5 and Figure 5 and Figure 6, the range corresponds the expected results. Only in air permeability warp rib weave is showing a somewhat higher air permeability very close to weft rib weave.

The results of air permeability obtained according to standard ISO 9237:1995—E are listed in Table 5 [40].

The last column in Table 5 presents the results of air permeability in (cm^3^/s, cm^2^) on a surface area of 50 cm^2^ at a pressure of 100 Pa according to the standard. Measurements at very low pressures up to 30 Pa were carried out in order to calculate the mean pore diameter of pores in fabrics which was assessed as problematic when microscopic fabric images were studied. 

This is confirmed by Figure 5 and Figure 6 with the accompanying equations and high coefficients of determination of R^2^. The calculation was done using Equation (4) by considering two different pore numbers. Firstly, as if all pores were equally distributed as the product of warp and weft density (Table 4, column 5). Secondly, as if only the pores between the threads with different interlacing were considered, and the pores between the threads with identical interlacing were not considered (Table 4, column 6). In this way, the number of pores was reduced because of an increase in the pore size that was considered, as seen in the figures of the examined fabrics themselves.

**Figure 7:**
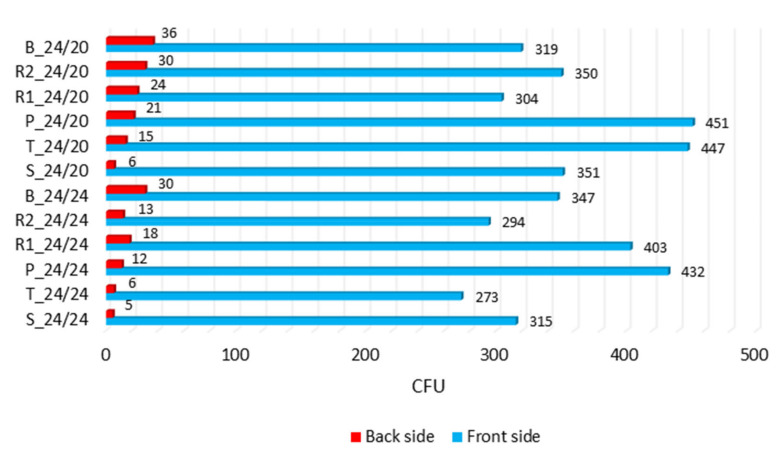
Results of microbial barrier permeability (after 24 h): The average number of bacterial colonies (CFU) on the front side and the back side.

**Figure 8:**
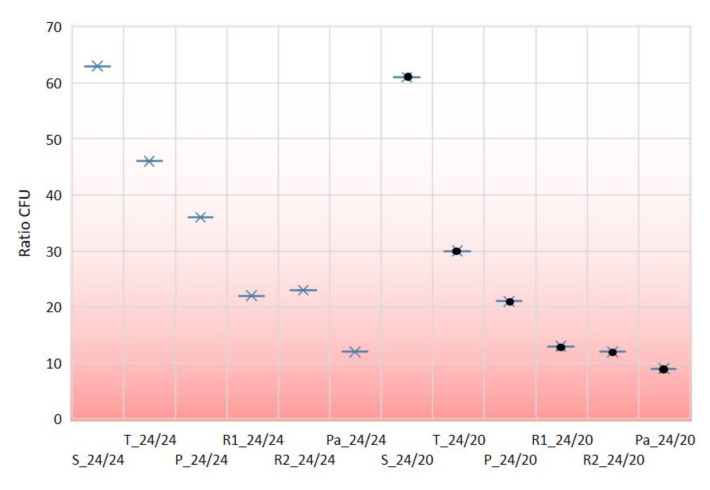
Ratio CFU—on the front side and on the back side. Quantity of microorganisms on fabric face as one microorganism was passed through the fabric.

**Figure 9:**
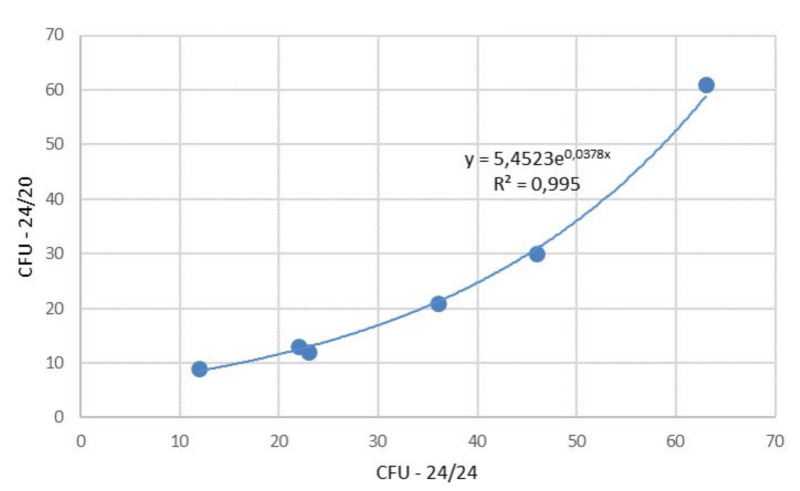
Values of Ratio CFU in correlation of densities 24/24 threads and 24/20 threads.

Table 6 and Figure 7, Figure 8 and Figure 9 present the results of microbial barrier properties of fabrics and permeability of microorganisms per weave patterns and densities. 

Comparing the number of microorganisms applied to the face of the fabric and the number of microorganisms that passed through to the fabric back side, a very similar trend in permeability of microorganisms in both fabric densities can be observed in Figure 7.

The permeability of microorganisms (Ratio CFU) is defined as the ratio of microbes (xx 1, Table 6) for one CFU that passed through the fabric (1) compared to the total number of CFUs (xx back side/front side). The Ratio CFU shows how many microorganisms must be applied to one side of the fabric in order for a microorganism to penetrate to the other side. The order of weaves in both densities was the same: satin, twill, plain, weft rib, warp rib, and basket (with one small exception between warp and weft rib where the order was changed presumably because of denting at density 24/20). It is important to indicate that the results of CFU do not follow the results of air permeability where the order was basket, ribs, satin, twill, and plain.

The redder area in Figure 8 indicates a higher permeability of microorganisms, i.e., a smaller microbial barrier. According to the results obtained (Ratio CFU) and the graphical representation in Figure 8, it can be claimed that the same order of the weave patterns remained from the best (most impermeable, upper asterisks) to the worst (most permeable, lower asterisks).

By increasing fabric density or weft density from 20 to 24 picks/cm, the values of Ratio CFU increased in all weave patterns which was confirmed with a relatively high correlation index of R^2^ = 0.995 (R = 0.997), Figure 9. 

The mutual differences in all examined parameters are observable in all fabrics woven in six different weave patterns and two thread densities 24/24 and 24/20. The highest specific fabric density, the smallest pore size and the smallest fabric thickness (plain weave) does not guarantee the largest microbial barrier. The satin weave fabric had the largest microbial barrier in both densities. It also had the greatest fabric thickness and greatest number of floating threads. The results revealed that floating threads had the greatest impact on the permeability of microorganisms.

The investigation results were statistically analyzed and are listed in Table 7. The correlations of each individual property of the examined fabrics with the permeability of bacteria are presented.

The correlation values listed in Table 7 reveal that it is appropriate to use the corrected number of pores (shown in sample figures, Figure 3) for the calculation of equivalent average diameter of pores and for the correlation with the bacterial penetration through the fabric. It is obvious that the porosity itself did not have any correlation with the permeability of bacteria because it gave only a fraction of the air in the fabric that can be composed of a small number of larger pores (R1, R2, and B) or a greater number of smaller pores. Sample thickness played a slightly larger role because it correlated positively with the permeability of bacteria—the greater the thickness the greater the number of bacteria rubbed for the permeability of one unit. Next, comes the mean equivalent pore diameter, which expectedly negatively correlated with the permeability of bacteria—larger pores, smaller number of rubbed bacteria for the passage of one unit. It is similar to the standard measurement of air permeability. Floating threads exhibited a slightly higher correlation. It should be also noted that the number of floating threads in a weave unit was taken as the sum of floating one warp thread and one weft thread (plain weave 1+1; twill weave 2+2; satin weave 4+4; rib weave R1 2+1; R2 1+2; and basket weave 2+2). The higher the number of floating threads, the higher the number of rubbed bacteria required for the penetration of one unit. The corrected number of pores surprisingly showed the highest single correlation. The correlation is positive, meaning that with a higher number of pores per unit area, on average, they are smaller, and more rubbed bacteria are required for the passage of one unit to the other side. 

The results revealed that one single fabric property did not have a predominant influence on the passage of bacteria through the fabric. Therefore, we had an idea to take three most significant factors as independent variables and to establish a correlation using multiple linear regression. Table 8, Table 9, and Table 10 show the values of multiple linear regression of fabrics with a density of 24/24 and a density of 24/20 and for all samples together.

The result for the samples with a density of 24/24 in the form of an equation is: *CFU = 0.0495·n_corr_ – 0.1268·d_e2_ + 4.7474·float + 11.6286 whereby the value of rss (Residual sum of Squares) = 7.870 and the value of the coefficient of determination R^2^ = 0.995 (R = 0.997).

The result for the samples with a density of 24/20 in the form of an equation is: *CFU = 0.0073·n_corr_ – 0.2357·d_e2_ + 7.2620·float + 31.8021, whereby the value of rss (Residual sum of Squares) = 12.381 and the value of the coefficient of determination R^2^ = 0.993 (R = 0.996).

The result for the samples with densities of 24/24 and 24/20 taken together in the form of an equation is: *CFU = 0.0245·n_corr_ - 0.2226·d_e2_ + 6.1697·float + 28.9004 whereby the values of rss (Residual sum of Squares) = 94.185 and the value of the coefficient of determination R^2^ = 0.976 (R = 0.988). 

The above presented results provide valuable information about the properties of the microbial barrier of fabrics, and they should be interpreted furthermore in order to give importance to the impact of weave pattern on their effectiveness in the use. Due to its alternating and maximum interlacing of warp and weft threads the plain weave gives the smallest fabric thickness, the greatest number of pores, but with smaller dimensions, which nevertheless allow relatively good permeability of microorganisms. Satin fabrics have a smooth and uniform surface, which is the result of only one interlacement of the warp and weft in a weave unit or every fifth crossing of the warp and weft is interlacing. In this case the yarn is more voluminous with larger air spaces in itself. Protruding fibers of the yarn create a compact fabric surface which makes it difficult for microorganisms to penetrate. 

The results show that even though the satin weave fabric had the high air permeability and the lowest specific mass it offered the greatest resistance to penetration of microorganisms compared to other weave patterns. It is known that a weave pattern with a higher number of floating threads (in this case satin weave) has higher air permeability than plain and twill weave patterns that have more frequent interlacements. Airflow separates the floating threads and creates larger pores in the fabric, allowing greater permeability, while the fabric in a relaxed state creates a more compact and impermeable structure. Since the objective of the work was to determine the effect of the weave pattern on the microbial barrier on the basis of the obtained research results, it can be claimed with certainty that the weave pattern significantly affects the properties of the microbial barrier, i.e., affects the permeability of microorganisms. More interlacements of warp and weft threads allow higher permeability of microorganisms, indicating that permeability of microorganisms depend on the pore size in the fabric in a relaxed state. Thus, the weave pattern creating a more voluminous fabric with less interlacing places creates a greater obstacle for the penetration of microorganisms in the direction of fabric cross-section.

From the previously published study by the authors of this paper, it can be concluded that antimicrobial aperture improves the properties of the microbial barrier, respectively, reducing the permeability of microorganisms by 11–37 % [45].

However, in the application of textile in medicine for the packaging of materials for sterilization and the separation of sterile from non-sterile space in operating rooms as well as for medical professional uniforms, water repellent aperture is not preferred. Water repellency is necessary when the bacteria are in wet environment. In many cases especially in cases of sterilization where the fluctuation of air and sterilizing media through the barrier is needed, water repellency does not contribute well. The permeability of microorganisms through the dry woven fabric is an unexplored area. According to previous researches of the woven fabric microbial barrier, the results have shown that the fabric woven in plain weave has higher permeability (ratio 32:1) than the fabric woven in twill 2/1 weave (ratio 60:1) [9].

For military woven fabrics, the results also showed the best microbial barrier for fabric woven in satin weave (Cotton/PA 6.6, 280 g/m^2^), which compared to fabric woven in twill 2/2 weave (cotton/PES, 250 g/m^2^) was impermeable to microorganisms. Weaves with frequent interlacements from the face to the back side of the fabric, in the weave pattern (plain, rib), create more expressive pores that allow easier penetration of microorganisms than weaves without sharp bindings (twill, satin). Twill 2/2 do not have abrupt and frequent sharp interlacements, but the warp and weft threads are arranged side by side in identical proportions, with gradual displacements [46]. 

According to the results of the research, it can be concluded that the change in the structure of fabric created by weave affects the permeability of microorganisms. Fabric porosity does not always correlate with permeability of microorganisms, therefore it is required to include other parameters in the research. The fabric weave that changes the interlacing of the weft and warp threads creates greater or lesser barriers for microorganisms. Warp and weft threads that interlace less (satin) create greater barrier for microorganisms, compared to the threads that interlace more frequently (plain). This research showed that more frequent and larger floating threads slow down the permeability of microorganisms through the fabric, which is the most interesting part of this investigation. Larger floating threads lead to increase in fabric thickness, which presents additional contribution in disabling the permeability of microorganisms through the fabric. 

## 4. Conclusions

This work dealt with studying the influence of fabric weave patterns on permeability of microorganisms. The results obtained lead to the following conclusions:

- The investigation results of microbial barrier permeability with respect to fabric weave pattern after extreme contamination with bacterial spores of *Geobacillus Stearothermophilus* and *Bacillus Atrophaeus* showed a significant influence of weave pattern on permeability of microorganisms. It was found that the difference between permeability of less bacteria permeable fabric (satin) and the most permeable fabric (basket) was in range of six to seven times. The assessment of the other fabrics put them in the same order regarding bacteria permeability.
- The correlation among the structural and physical characteristics and obtained results of microbial permeability confirmed that neither sample had a predominant influence. The most influential element of weave on permeability is corrected number of pores, their size and floating of warp and weft yarns. As expected, the number of pores and the number of floating yarns correlated positively and equivalent average size correlated negatively with CFU.
- Multiple linear regression of pore numbers, equivalent average pore diameter and floating threads produced equations which correspond in 99% to the measuring results for densities 24/24 and 24/20, and more than 98% when both densities of the set were taken into consideration. It gives the possibility to predict bacteria penetration through woven fabrics without significant differences in densities of warp and weft—woven structures near to square woven fabrics (up to ratio density of weft/ density of warp = 0.8).
- It was found that the floating threads have significant influence on the permeability of microorganisms. Without the effect of floating threads, such high correlation between multiple regression results and CFU could not be recognized.
- With both thread densities (24/24 and 24/20) the sequence of permeability per weave patterns was the same: satin, twill, plain, rib (R1, R2), and basket.
- The accuracy of the results is confirmed by behavior of rib weaves (R1 and R2) which are the same structure but turned by 90° and gave almost identical results.

Considering the objective of this work, it can be concluded that the elements of weave which dominantly influence bacteria penetration through the fabric in dry conditions, were successfully identified. Those dominant elements are: the number of pores among yarns that consecutively interlace differently, their equivalent average diameter and the number of floating warp and weft yarns.

Understanding this fact and taking into an account other constructional, technological and after-treatment characteristics, the constructors of woven fabrics are given an opportunity to produce woven fabrics for the use in medical and sterilization area. They can also be used in all sterile areas for packaging food, medications, etc. It is also important to note that the satin weave easily enables a significant increase in density, which increases the number of pores, significantly reduces the size of pores, and consequently increases the barrier effect of the fabric on bacterial penetration.

## Figures and Tables

**Table 1:** Impact of fabrics’ constructional parameters on pore size [34].

Degree of Influence on Pore Size	Source of Influence			
	Yarn Fineness (tex)	Filament Fineness (dtex)	Yarn Density (Threads/cm)	Weave Type
Micro pores (<10^−3^ m)	+	+++	++	+
Meso pores (10^−3^–10^−2^ m)	+++	+	+++	+++

+++ very important, ++ important, + not very important.

**Table 2:** Constructional/physical parameters of samples with density 24/24 threads/cm.

Sample	Label		Mass per Area (g/m^2^)	Thickness (mm)	Linear Density (tex)		Fabric Density (Threads/cm)	
					Warp	Weft	Warp	Weft
Satin S4/1(2)	S_24/24	Mean	177.1	0.398	34.5	35.6	24.6	23.9
		SD	1.1	0	1.6	1.9	0.8	0.6
		CV (%)	0.6	1.2	4.8	5.4	3.4	2.4
Twill T2/2 S	T_24/24	Mean	182.3	0.362	34.2	35.8	24.8	24.1
		SD	2.5	0	2.1	1.8	0.4	0.3
		CV (%)	1.4	1.2	6.1	5.0	1.7	1.3
Plain P1/1	P_24/24	Mean	186.7	0.332	34.2	36.5	24.2	24.0
		SD	0.2	0	0.7	0.9	0.4	0
		CV (%)	0.1	1.1	2.1	2.6	1.7	0
Rib 1/1 (2+2)	R1_24/24	Mean	177.1	0.377	35.1	36.7	24.5	24.0
		SD	1.2	0	1.0	0.8	0.5	0.8
		CV (%)	0.7	0.9	2.8	2.3	2.2	3.4
Rib 2/2 (1+1)	R2_24/24	Mean	178.1	0.376	35.9	35.6	24.40	24.3
		SD	1.6	0	1.1	1.0	0.5	0.5
		CV (%)	0.9	1.2	3.2	2.9	2.1	2.0
Basket B2/2	B_24/24	Mean	178.8	0.379	35.3	36.3	24.3	23.7
		SD	1.1	0	1.6	1.1	0.5	0.5
		CV (%)	0.6	0.9	4.4	3.1	2.0	2.0

SD: standard deviation; CV: coefficient variation (%); P: plane weave; T: twill weave; S: Satin weave; B: basket weave; R: rib weave.

**Table 3:** Constructional/physical parameters of samples with density 24/20 threads/cm.

Sample	Label		Mass per Area (g/m^2^)	Thickness (mm)	Linear Density (tex)		Fabric Density (Threads/cm)	
					Warp	Weft	Warp	Weft
Satin S4/1(2)	S_24/20	Mean	161.2	0.370	34.7	36.0	24.3	19.7
		SD	1.6	0	1.2	1.3	0.5	0.5
		CV (%)	1.0	0.8	3.4	3.7	2.0	2.5
Twill T2/2 S	T_24/20	Mean	162.0	0.357	34.7	35.3	24.4	19.9
		SD	2.0	0	2.4	1.7	0.5	0.6
		CV (%)	1.2	0.6	6.8	4.7	2.1	2.9
Plain P1/1	P_24/20	Mean	165.8	0.333	35.7	35.6	24.3	20.3
		SD	0.5	0	1.5	1.2	0.5	0.5
		CV (%)	0.3	1.1	4.3	3.3	2.0	2.4
Rib 1/1 (2+2)	R1_24/20	Mean	162.9	0.364	34.0	36.3	24.4	19.6
		SD	1.3	0	1.3	1.4	0.5	0.5
		CV (%)	0.8	1.3	3.9	3.8	2.1	2.6
Rib 2/2 (1+1)	R2_24/20	Mean	160.4	0.363	35.3	36.6	23.5	19.9
		SD	0.8	0	1.5	1.7	0.5	0.3
		CV (%)	0.5	1.9	4.2	4.6	2.2	1.6
Basket B2/2	B_24/20	Mean	161.3	0.364	34.1	36.0	24.4	19.6
		SD	0.6	0	2.3	2.1	0.5	0.7
		CV (%)	0.4	1.6	6.7	5.9	2.1	3.6

SD: standard deviation; CV: coefficient variation (%); P: plane weave; T: twill weave; S: Satin weave; B: basket weave; R: rib weave.

**Table 4:** Results of measuring physical characteristics of samples.

Sample	Specific Density (ρ_fabric_, g/cm^3^) ^1^	Fabric Porosity (P, %) ^2^	Set Number of Pores (n)	Actual Number of Pores (n) ^3^	Corrected Number of Pores (n)	EAD Diameter of Pores 1 (μm)	EAD Diameter of Pores 2 (µm)
S_24/24	0.445	70.7	576	588	588	122.20	122.20
T_24/24	0.504	66.9	576	598	598	119.70	119.70
P_24/24	0.562	63.0	576	581	581	97.50	97.50
R1_24/24	0.470	69.1	576	588	294	126.70	150.50
R2_24/24	0.474	68.8	576	593	297	126.50	150.40
B_24/24	0.472	69.0	576	576	144	135.90	192.14
S_24/20	0.436	71.3	480	479	479	141.50	141.50
T_24/20	0.454	70.1	480	486	486	133.90	133.90
P_24/20	0.498	67.2	480	493	493	130.45	130.45
R1_24/20	0.448	70.6	480	478	239	152.01	180.77
R2_24/20	0.442	70.9	480	468	234	154.13	183.29
B_24/20	0.443	70.8	480	478	120	157.90	223.30

EAD: Equivalent average diameter of pores.

**Table 5:** Results of air permeability of samples (ISO 9237:1995 -E).

Sample	Air Permeability (cm^3^/s, cm^2^)–Measurement Area 50 cm^2^						Values of Coefficient *A* in Equation (3)
	Pressure (Pa)						
	10 Pa	15 Pa	20 Pa	25 Pa	30 Pa	100 Pa	
S_24/24	4.2	6.5	8.7	10.9	13.6	41.77	0.4418
T_24/24	4.2	6.7	9.0	11.4	13.8	38.71	0.4540
P_24/24	1.9	3.1	4.2	5.3	6.5	21.77	0.2120
R1_24/24	5.4	8.1	10.9	13.4	16.0	44.14	0.5371
R2_24/24	5.6	8.2	11.0	13.4	16.1	42.93	0.5409
B_24/24	7.5	11.2	14.4	17.1	20.2	51.51	0.6953
S_24/20	6.8	10.6	13.9	17.5	20.8	60.88	0.6962
T_24/20	5.6	8.5	11.7	14.4	17.4	52.09	0.5776
P_24/20	5.8	8.8	11.6	14.2	17.2	45.52	0.5747
R1_24/20	10.1	15.1	19.2	23.0	27.6	76.49	0.9398
R2_24/20	10.4	15.4	19.9	24.0	28.4	73.29	0.9711
B_24/20	12.1	17.9	22.1	26.7	32.1	78.33	1.0942

**Table 6:** Results of microbial barrier permeability for the tested textiles after extreme contamination with bacterial spores *Geobacillus stearothermophilus* and *Bacillus atrophaeus*.

Sample	Number of Isolates	Colonies of the Back Side/Plate Mean ± SD (range)	The Average Number of Colonies on the Front Side	Ratio CFU (on the Front Side and on the Back Side)
S_24/24	12	5 ± 3.0 (1–8)	315	63:1
T_24/24	12	6 ± 2.3 (3–10)	273	46:1
P_24/24	12	12 ± 8.9 (5–28)	432	36:1
R1_24/24	12	18 ± 8.3 (9–25)	403	22:1
R2_24/24	12	13 ± 3.3 (7–22)	294	23:1
B_24/24	12	30 ± 24.9 (9–56)	347	12:1
S_24/20	12	6 ± 8.2 (1–18)	351	61:1
T_24/20	12	15 ± 6.0 (9–21)	447	30:1
P_24/20	12	21 ± 10.6 (10–31)	451	21:1
R1_24/20	12	24 ± 21.6 (4–47)	304	13:1
R2_24/20	12	30 ± 21.1 (8–50)	350	12:1
B_24/20	12	36 ± 22.7 (17–61)	319	9:1

CFU: Colony Forming Unit.

**Table 7:** Correlation values of individual fabric properties and the number of necessary rubbed bacteria for the passage of one unit with thread densities of 24/24 threads/cm and 24/20 threads/cm.

Sample	Actual Number of Pores (n)^3^	Corrected Number of Pores (n)	EAD Diameter of Pores 1 (μm)	EAD Diameter of Pores 2 (d_e2_, µm)	Fabric Porosity (P, %)	Thickness (mm)	Floating (yarns)	Air Permeability (cm^3^/s, cm^2^)	CFU
S_24/24	588	588	122.20	122.20	70.7	0.40	8	41.77	63
T_24/24	598	598	119.70	119.70	66.9	0.36	4	38.71	46
P_24/24	581	581	97.50	97.50	63.0	0.33	2	21.77	36
R1_24/24	577	294	126.70	150.50	68.8	0.38	3	44.14	22
R2_24/24	588	297	126.50	150.40	69.0	0.38	3	42.93	23
B_24/24	593	144	135.90	192.14	69.1	0.38	4	51.51	12
S_24/20	479	479	141.50	141.50	71.3	0.37	8	60.88	61
T_24/20	486	486	133.90	133.90	70.1	0.36	4	52.09	30
P_24/20	493	493	130.45	130.45	67.2	0.33	2	45.52	21
R1_24/20	478	239	152.01	180.77	70.9	0.36	3	76.49	13
R2_24/20	466	234	154.13	183.29	70.8	0.36	3	73.29	12
B_24/20	478	120	157.90	223.30	70.6	0.36	4	78.33	9
**corr; 1-6; 7-12**	−0.05477	0.99859	0.87163	0.97863	0.97403	0.97272	1	0.85553	0.95338
**corr; 1-6; CFU 1-6**	0.17785	0.88813	−0.40059	−0.72543	0.04035	0.14203	0.69053	−0.36169	
**corr; 7-12; CFU 7-12**	0.20376	0.70413	−0.40990	−0.62224	0.19370	0.23062	0.86498	−0.44411	
**corr; 1-12; CFU 1-12**	0.28234	0.80833	-0.41593	-0.69669	-0.04881	0.24264	0.75298	-0.45598	

corr: Correlation Coefficient; EAD: Equivalent average diameter of pores.

**Table 8:** Multiple linear regression of fabric samples with a density of 24/24.

Sample	Corrected Number of Pores (n_corr_)	EAD Diameter of Pores 2 (d_e2_, µm)	Number of Float (yarns)	CFU	Calculated *CFU	Error
S_24/24	588	122.20	8	63	63.22	0.22
T_24/24	598	119.70	4	46	45.04	0.96
P_24/24	581	97.50	2	36	37.52	1.52
R1_24/24	294	150.50	3	22	21.34	0.66
R2_24/24	297	150.40	3	23	21.50	1.50
B_24/24	144	192.14	4	12	13.58	1.38

EAD: Equivalent average diameter of pores.

**Table 9:** Multiple linear regression of samples with a density of 24/20.

Sample	Corrected Number of Pores (n_corr_)	EAD Diameter of Pores 2 (d_e2_, µm)	Number of Float (yarns)	CFU	Calculated *CFU	Error
S_24/20	479	141.50	8	61	60.05	0.95
T_24/20	486	133.90	4	30	32.84	2.84
P_24/20	493	130.45	2	21	19.18	1.82
R1_24/20	239	180.77	3	13	12.73	0.27
R2_24/20	234	183.29	3	12	12.10	0.10
B_24/20	120	223.30	4	9	9.10	0.10

EAD: Equivalent average diameter of pores.

**Table 10:** Multiple linear regression of samples with densities of 24/24 and 24/20 taken together.

Sample	Corrected Number of Pores (n_corr_)	EAD Diameter of Pores 2 (d_e2_, µm)	Number of Float (yarns)	CFU	Calculated *CFU	Error
S_24/24	588	122.20	8	63	65.44	2.44
T_24/24	598	119.70	4	46	41.57	4.43
P_24/24	581	97.50	2	36	33.75	2.25
R1_24/24	294	150.50	3	22	21.11	0.89
R2_24/24	297	150.40	3	23	21.20	1.80
B_24/24	144	192.14	4	12	14.34	2.34
S_24/20	479	141.50	8	61	58.48	2.52
T_24/20	486	133.90	4	30	35.67	5.67
P_24/20	493	130.45	2	21	24.27	3.27
R1_24/20	239	180.77	3	13	13.02	0.02
R2_24/20	234	183.29	3	12	12.34	0.34
B_24/20	120	223.30	4	9	6.82	2.18

EAD: Equivalent average diameter of pores.
